# Predictors of health professionals’ satisfaction with continuing education: A cross-sectional study*

**DOI:** 10.1590/1518-8345.3637.3315

**Published:** 2020-07-01

**Authors:** Francisco Javier Muñoz-Castro, Eloisa Valverde-Gambero, Manuel Herrera-Usagre

**Affiliations:** 1 Agencia de Calidad Sanitaria de Andalucía, Consejería de Salud, Sevilla, Andalucía, Spain.; 2 Universidad Pablo de Olavide, Agencia de Calidad Sanitaria de Andalucía, Consejería de Salud, Sevilla, Andalucía, Spain.

**Keywords:** Education, Continuing, Personal Satisfaction, Education, Distance, Students, Health Occupations, Staff Development, Clinical Conference, Educação Continuada, Satisfação Pessoal, Educação a Distância, Estudantes de Ciências da Saúde, Desenvolvimento de Pessoal, Conferência Clínica, Educación Continua, Satisfacción Personal, Educación a Distancia, Estudiantes del Área de la Salud, Desarrollo de Personal, Conferencia Clínica

## Abstract

**Objectives:**

to verify which organizational, methodological, and resource-related characteristics of Continuing Health Education (CHE) help to best predict the professionals´ satisfaction.

**Method:**

a cross-sectional study with multivariate logistic regressions to predict a high mean satisfaction with different dimensions of educational actions used: Overall satisfaction, Utility, Methodology, Organization and resources, and Teaching Capacity. 25,281 satisfaction questionnaires have been analysed completed by health professionals attending 1,228 training activities in Andalusia (Spain), during the period from March 2012 to April 2015.

**Results:**

the characteristics that best predict a high overall satisfaction are the following: clinical session type as opposed to the workshop (Odds Ratio [OR]=2.07, p<0.001); face-to-face attendance modality (OR=3.88, p<0.001) or semi-personal-attendance (OR=2.83, p<0.001), as opposed to e-learning; and 1-2 days in duration (OR=2.38, p<0.001) as opposed to those of between 3 and 14 days. A lower number of hours (OR=0.99, p<0.001) and a lower number of professionals (OR=0.98, p<0.05) also increase the probability. Having the educational actions accredited increases the probabilities in the following dimensions: Utility (OR=1.33, p<0.05), Methodology (OR=1.5, p<0.01) and Teaching capacity (OR=1.5, p<0.01).

**Conclusion:**

the study provides relevant information on aspects that improve professional satisfaction, such as that e-learning activities should improve their content, teaching methods, and styles, or that face-to-face clinical sessions are the type of CHE with the greatest satisfaction.

## Abstract (pt)

**Objetivo:**

verificar quais características organizacionais, metodológicas e relacionadas a recursos da educação continuada em saúde (ECS) ajudam a predizer melhor a satisfação dos profissionais.

**Método:**

estudo transversal com regressões logísticas multivariadas para predizer uma alta satisfação média com diferentes dimensões das ações educativas utilizadas: Satisfação Geral, Utilidade, Metodologia, Organização e Recursos, e Capacidade de Ensino. Foram analisados 25.281 questionários de satisfação preenchidos por profissionais de saúde que participaram de 1.228 atividades de treinamento na Andaluzia (Espanha), no período de março de 2012 a abril de 2015.

**Resultados:**

características que melhor predizem uma alta Satisfação Geral são as seguintes: Método de sessão clínica em oposição a *workshops* (*Odds* *Ratio*[OR]=2,07;p<0,001); modalidade presencial (OR=3,88;p<0,001) ou semipresencial (OR=2,83;p<0,001) em oposição a *e-learning*; e 1-2 dias de duração (OR=2,38;p<0,001) em oposição a 3-14 dias. Um menor número de horas (OR=0,99;p<0,001) e de profissionais (OR=0,98;p<0,05) também aumenta a probabilidade. A acreditação das ações educativas aumenta as probabilidades nas dimensões: Utilidade (OR=1,33;p<0,05), Metodologia (OR=1,5;p<0,01) e Capacidade de Ensino (OR=1,5;p<0,01).

**Conclusão:**

o estudo fornece informações relevantes sobre aspectos que melhoram a satisfação profissional, como a opinião de que atividades *e-learning* precisam melhorar seu conteúdo, métodos e estilos de ensino, ou de que sessões clínicas presenciais são o tipo de ECS com maior satisfação.

## Abstract (es)

**Objetivo:**

verificar qué características organizativas, metodológicas y de recursos de la Educación Continua en Salud (ECS) ayudan a predecir mejor la satisfacción de los profesionales.

**Método:**

se utiliza un estudio transversal con regresiones logísticas multivariadas para predecir un alto promedio de satisfacción con los diferentes tipos de acciones educativas: Satisfacción general, Utilidad, Metodología, Organización y recursos y Capacidad docente. Se han analizado 25.281 cuestionarios de satisfacción respondidos por los profesionales de la salud que asistieron a 1228 actividades formativas, en Andalucía (España), durante el período de marzo a abril de 2012.

**Resultados:**

características que mejor predicen una alta Satisfacción general: Tipo de sesión clínica frente al taller (*Odds*Ratio [OR]=2,07, p<0,001); modalidad de asistencia presencial (OR=3,88, p<0,001) o semipersonal (OR=2,83, p<0,001), frente al aprendizaje electrónico (*e-learning*); y 1-2 días de duración (OR=2,38, p<0,001) frente a 3-14 días. Una menor cantidad de horas (OR=0,99, p<0,001) y una menor cantidad de profesionales (OR=0,98, p<0,05) también aumentan la probabilidad. Acreditar las acciones educativas aumenta las probabilidades en las siguientes dimensiones: Utilidad (OR=1,33, p<0,05), Metodología (OR=1,5, p<0,01) y Capacidad docente (OR=1,5, p<0,01).

**Conclusión:**

el estudio aporta información relevante sobre aspectos que mejoran la satisfacción profesional, como que las actividades de aprendizaje electrónico deben mejorar sus contenidos, métodos y estilos de enseñanza o que las sesiones clínicas personales son el tipo de ECS con mayor satisfacción.

## Introduction

Continuing education is the group of educational activities designed to maintain, develop or increase knowledge, skills, and the performance of active professionals^(^ 1 ^)^, with it therefore being differentiated from other stages of education, such as degree education, specialized education and post-graduate education.

This is an essential and strategic tool to improve quality in health systems^(^ 2 ^)^, to improve results in the health of patients^(^ 3 ^-^ 4 ^)^, to satisfy health professionals^(^ 5 ^-^ 6 ^)^, and to transfer and exchange knowledge^(^ 7 ^-^ 8 ^)^.

Various systems of accreditation of continuing education have been developed, at the international level and addressed towards health professionals^(^ 9 ^-^ 11 ^)^, in order to guarantee the quality level of the educational actions that are performed, by means of the periodical updating and continuous improvement of models^(^ 12 ^-^ 13 ^)^.

Since the year 2003, the Andalusian Agency for Healthcare Quality (*Agencia de Calidad Sanitaria de Andalucía*, ACSA) has implemented a model for accreditation of continuing education activities of health professionals^(^ 14 ^)^, in consonance with the quality strategies of the Andalusian Health System^(^ 15 ^)^ and with the National Continuing Education Committee criteria. It is structured in quality elements related to aspects such as the need that justifies the education, the characteristics of the teaching-learning process that is to be carried out, the profile of the professionals participating in the education, the resources used in its planning and development, and the results of the educational process. The assessment of these elements determines the degree of quality of the education designed, and, with this, the accreditation or non-accreditation of the educational action in question.

In addition, within the educational activities that are performed, there are different modalities of education depending on the degree of personal attendance of the participants, “face-to-face learning”: with physical presence of the professionals, “blended learning”: it integrates the characteristics inherent to personal-attendance and non-personal-attendance without any virtual tools, “e-learning”: carried out via the new virtual communication tools; and different typologies, taking into consideration the characteristics of the educational process of educational activities^(^ 16 ^)^: “courses”: educational activities addressed towards the acquisition of knowledge, “workshops”: practical educational activities to develop skills, “clinical conferences”: periodical activities in which to exchange scientific information, opinions, and experiences on the commonalities of the daily practice.

The choice of these will depend, on one hand, on the detection of educational needs, and, on the other, on the learning objectives that may have been established and, finally, on the resources available for conducting the educational activities.

In recent years, there has been an exponential increase in education in the health area, supported by new technologies, also known as e-learning, internet-based learning, online learning, computer-assisted learning, or web-based learning^(^ 17 ^)^. Various studies have indicated the advantages of this educational modality among health professionals, such as the flexibility of self-paced learning, adapted to the timings of the professionals, the plasticity to different styles of learning, the permanent availability of access to contents, the capacity to overcome time-related hurdles, the costs of travelling, the limitations of physical spaces for teaching, and the difficulties faced by professionals in rural environments distant from large educational centres^(^ 17 ^-^ 20 ^)^. In addition, international models of accreditation of continuing education consider specific standards for the modality of e-learning^(^ 21 ^)^.

Documents such as *Training Evaluation Field Guide: Demonstrating the Value of Training at Every Level* ^(^ 22 ^)^ recommend integrating various systems of evaluation into the design of educational activities, which may allow for the results achieved to be analysed. For this purpose, various models of evaluation of activities exist, the Kirkpatrick model^(^ 23 ^)^ being one of the most widely used.

The Kirkpatrick model proposes evaluation of education on 4 levels: Reaction/Satisfaction, Learning, Behaviour/Transfer, and Results/Impact. The use of this model has grown to cover the evaluation of continuing education activities, as demonstrated in numerous studies^(^ 24 ^-^ 25 ^)^. In order to facilitate standardized instruments for users that allow for the evaluation of given aspects related to the design and development of the educational activities carried out, ACSA created a tool named “eValúa”^(^ 26 ^)^, in which various online questionnaires are included and which allows for the data collected through them to be processed.

The questionnaire on the satisfaction of professionals included in eValúa is a tool that has been validated^(^ 27 ^)^ and developed taking into account the criteria established in the ACSA accreditation programmes^(^ 28 ^)^ that is designed to provide a reply on level 1 of the Kirkpatrick evaluation model (reaction level). This questionnaire considers 23 items, grouped together in five dimensions (Utility, Methodology, Organization and resources, Teaching capacity, and Overall valuation), and other descriptive aspects of the educational activity, including the typology, the modality, and the number of professionals.

Taking some studies into account^(^ 29 ^)^, the educational modality used (face-to-face learning, blended learning, e-learning and b-learning) and the proportion of the number of professionals/teachers in the activities, have a lineal relationship with the quality of the education perceived. The main objective of this article is to verify which organizational, methodological, and resource-related characteristics of continuing health education help to best predict the professionals´ satisfaction.

## Method

For this study, the Questionnaire on the Satisfaction of Professionals (QSP) has been used, with professionals who are experts in the quality of education, belonging to ACSA and to the Units of Education or management of knowledge of other entities of the Public Health System of Andalusia, having intervened in its preparation. For the preparation thereof, various questionnaires used to measure the degree of satisfaction of the participants in educational activities in the area of health were analysed and, based on this information, the dimensions were designed and the items were prepared. The psychometric properties of reliability and validity of the questionnaire have already been analysed, and improvements have been incorporated^(^ 27 ^)^.

The study group consists of a random, non-stratified sample of educational activities addressed towards health professionals (physicians, nurses, pharmacists, and other health care professionals), in any centres in the region of Andalusia (Spain), during the period from March 2012 to April 2015, in which the *eValúa* QSP was used to explore the level of satisfaction of the participants with each activity (n=1,228 educational activities; N=25,281 professionals who have answered the QSP).

The evaluation of the satisfaction level must be carried out as from the day on which the educational action finalizes, at which moment the *eValúa* tool activates the corresponding link for the web version of the questionnaire. As from that date, the person responsible for the education or for coordinating the educational activity sends an e-mail from the application to all the professionals, with the information necessary to complete the questionnaire: the web link, the term, details on the activity, the purpose of the survey, and privacy rules. Alternatively, they may print the questionnaire out in paper format and hand it in by hand, on finalizing the education, having, subsequently, to introduce the results manually for the processing of the data.

*Dependent Variables:* All of the evaluation items of the QSP are shown on a scale on which 0 is the “lowest degree of satisfaction, or total disagreement” and 10 is the “highest degree of satisfaction, or total agreement”. The mean scores of all of the items contained in each one of the dimensions of the questionnaire that the professionals have to complete after receiving the education have been calculated: *Overall satisfaction* (2 items, Cronbach’s alpha=0.964), *Utility* (3 items, Cronbach’s alpha=0,913), *Methodology* (6 items, Cronbach’s alpha=0,95), *Organization and resources* (6 items, Cronbach’s alpha=0,923), and *Teaching capacity* (5 items, Cronbach’s alpha=0,972) (Table 3). Given that the dependent variables (DVs) of the model did not comply with part of the criteria to do a least squares regression (homoscedasticity, residual normality, and autocorrelation), it was decided to dichotomize the DVs to be able to launch five logistic regressions. Due to high mean scores in all of the DVs, all of the educational activities that are over the median have been considered as “high scores” while those that are below the median were classified as “low scores”. Therefore, the logistic regressions try to predict a “high score” using the independent variables.

**Table 1:** Descriptive statistics. Andalusia, Spain, 2012-2015

Numeric variables	Mean	Typical deviation	Minimum	Maximum	Median
Number of professionals who have attended	27	24	1	500	24
Number of hours of duration	11	21	1	301	3
Overall Mean	7.89	1.06	2.00	10.00	8.05
Utility Mean	8.39	0.91	2.50	10.00	8.50
Methodology Mean	8.39	0.92	2.00	10.00	8.53
Organization and resources Mean	8.39	0.86	3.80	10.00	8.50
Teaching capacity Mean	8.61	0.81	3.50	10.00	8.70
**Categorical variables**			**Count**		**%**
Accredited education	No		208		16.9%
	Yes		1,020		83.1%
Type of education	Workshop		166		13.8%
	Course		462		38.3%
	Clinical session		579		48.0%
Modality of the education	Face-to-face learning		853		70.1%
	E-Learning		111		9.1%
	Blended learning		252		20.7%
Duration in days	1 to 2 days		738		60.1%
	3 to 14 days		201		16.4%
	15 to 50 days		230		18.7%
	More than 51 days		59		4.8%

**Table 2:** Correlations between the variables included in the study. Andalusia, Spain, 2012-2015

	Number of professionals	Duration (hours)	Duration (days)	Overall	Utility	Methodology	Organization and resources	Teaching capacity
Number of professionals who have attended	1							
Number of hours of duration	0.001	1						
Duration of the education (no. of days)	0.008	0.064*	1					
Overall Mean	-0.096^†^	-0.081^†^	-0.026	1				
Utility Mean	-0.098^†^	-0.033	0.001	0.750^†^	1			
Methodology Mean	-0.109^†^	-0.045	0.010	0.798^†^	0.878^†^	1		
Organization and resources Mean	-0.076^†^	-0.053	0.000	0.744^†^	0.774^†^	0.841^†^	1	
Teaching capacity Mean	-0.125^†^	-0.014	0.022	0.686^†^	0.841^†^	0.831^†^	0.710^†^	1

* The correlation is significant at the 0.05 level;

† The correlation is significant at the 0.01 level

**Table 3:** Logistic Regression models with high ratings of continuing health education courses. Andalusia, Spain, 2012-2015

	Odds Ratio (95% CI)				
	Overall	Utility	Methodology	Organization and resources	Teaching capacity
Accredited activity	1.321 (0.923-1.895)	1.328*(0.952-1.858)	1.502^†^ (1.064-2.128)	1.291(0.931-1.796)	1.502^†^ (1.072-2.106)
Workshop type	1	1	1	1	1
Course type	0.499^‡^ (0.330-0.755)	0.844 (0.564-1.263)	1.020 (0.673-1.552)	0.925(0.622-1.380)	1.225(0.803-1.862)
Session type	2.075^‡^ (1.355-3.175)	1.217 (0.802-1.847)	3.148^‡^ (2.053-4.868)	2.105^‡^ (1.399-3.189)	0.657*(0.424-1.010)
E-learning	1	1	1	1	1
Face-to-face learning	3.878^‡^ (2.005-7.892)	2.556^‡^ (1.424-4.704)	2.824^‡^ (1.518-5.407)	1.100(0.634-1.920)	3.547^‡^ (1.992-6.451)
Blended learning	2.830^‡^ (1.493-5.678)	2.833^‡^ (1.618-5.109)	3.449^‡^ (1.894-6.515)	1.351(0.806-2.288)	4.394^‡^ (2.538-7.784)
From 1 to 2 days	1	1	1	1	1
From 3 to 14 days	0.420^‡^ (0.259-0.675)	1.101 (0.707-1.715)	1.338(0.851-2.110)	0.945(0.609-1.465)	0.870(0.547-1.387)
From 15 to 50 days	0.813 (0.484-1.372)	0.888 (0.538-1.467)	0.756(0.449-1.271)	0.787(0.478-1.291)	0.714(0.424-1.199)
More than 51 days	1.712 (0.732-4.082)	1.220 (0.540-2.784)	1.207(0.521-2.791)	0.828(0.365-1.833)	1.161(0.497-2.836)
No. of professionals	0.986^‡^ (0.975-0.996)	0.987^†^ (0.977-0.997)	0.991*(0.981-1.001)	0.994(0.985-1.003)	0.985^‡^ (0.975-0.995)
No. of hours	0.988*(0.973-0.999)	0.996 (0.984-1.007)	1.001(0.989-1.012)	1.002(0.992-1.013)	1.000(0.989-1.013)
Constant	0.409^†^ (0.168-0.964)	0.427^†^ (0.191-0.941)	0.186^‡^ (0.079-0.429)	0.556(0.260-1.179)	0.537(0.240-1.190)
N	1,192	1,107	1,106	1,143	1,107
Nagelkerke Pseudo-R2	0.321	0.055	0.143	0.062	0.081
AUC	0.784	0.602	0.680	0.618	0.619

* p-value <0.1;

† p-value <0.05;

‡ p-value <0.01

*Independent Variables:* Each one of the activities included is shown with the following additional information to the evaluation: *Number of professionals who have attended* (continuous variable), *Number of hours of duration of the educational action* (continuous variable), *Duration in days of the education* (categorical variable: “1 to 2 days”, “3 to 14 days”, “15 to 50 days”, “More than 51 days”); *Modality of the activity* (categorical variable: “Face-to-face learning”, “*e-learning”* or “Blended learning”); *Typology of the educational action* (categorical variable: “course”, “workshop”, “clinical conference”); *Activity accredited by ACSA* (dichotomic variable: “yes” or “no”).

Firstly, a correlation analysis was performed between all of the non-categorical variables included in the study (Spearman’s rank correlation coefficient). Subsequently, five logistic regression analyses were performed with each one of the dichotomized satisfaction dimensions included in the QSP to verify which independent or explicative variables are the ones that have the most influence on there being a high level of satisfaction of professionals with the educational activity that they have received. For this, the R statistic package has been used. The Odds Ratio (OR) and its 95% confidence intervals were used. In addition to Nagelkerke pseudo-R-squared, the area under the Receiver Operating Characteristic (ROC) curve, or the capacity of the model to correctly classify the educational activities with high satisfaction (Area Under the Curve, AUC) have been included.

## Results

In Table 1, the descriptive statistics are shown, both for the dependent and the independent variables. The mean number of attendees for the training activities was 27 with a high typical deviation (24) showing a wide range of training activities sizes. The mean duration was 11 hours but ranged between a minimum of 1 hour and a maximum of 301 hours, thus showing a very high typical deviation (21). Teaching capacity was the dimension that showed the highest mean score (8.61) while the Overall satisfaction indicator showed the lowest (7.89). Regarding the other characteristics of the training activities, 60% of them took place in 1 or 2 days, while another 35% took between 3 to 50 days. Close to 5% of the training activities used in this study lasted more than 51 days. The majority of those training activities were Physical (70%) while only 9% were e-learning and nearly 21% required a blended learning modality, which is to say, blending physical activities and e-learning activities. Almost half of the activities were organized as clinical sessions in a health care setting. Another 14% of the training activities were organized as workshops while 38% were specialized courses. Finally, with regard to the accreditation status, only 17% of the activities were accredited through the ACSA Accreditation Programme.

In Table 2, the correlations between the numerical variables included in the study are shown. The first thing to be highlighted is the negative correlation between the number of professionals and the mean satisfaction level in all of the dimensions of the QSP. In spite of the fact that these negative correlations are statistically significant, the correlation coefficients are very low. The existence of a low but statistically significant correlation between the number of hours of educational activity and a low level of overall satisfaction is to be highlighted. On addressing the correlations between the different dimensions of the questionnaire, two aspects stand out: firstly, satisfaction with the teaching capacity is the dimension that has the least correlation with the overall satisfaction of the educational activity. Secondly, the high correlation between the Methodology and Utility dimensions stands out. In the rest of the correlations between dimensions, high levels are maintained with coefficients of over 0.7.

In Table 3, a set of five logistic regressions are shown, one for each one of the dimensions that are valued in the questionnaire. The objective is to verify which characteristics have the most influence on predicting a high mean satisfaction level with the health education activity received by the professionals. With the exception of the Overall valuation (32.1%) and of the evaluation of Methodology (14.3%), the logistic regression models performed showed percentages of variance taken to be moderate, as well as a uniform performance in sensitivity analysis (AUC). This is the case of the models performed with the evaluation of Utility (5.5%), Organization and resources (6.2%), and Teaching capacity (8.1%).

The Clinical session or conference type of education shows ratings that are considerably higher in workshops in all categories, with the exception of Teaching capacity. The sessions show twice the probability of workshops to be highly valued overall and, specifically, with respect to the Organization and resources used, and three times with respect to the Methodology used. However, the sessions show 52% fewer probabilities of Teaching capacity being highly valued with respect to workshops (OR=0.657). The workshops are twice as likely as courses to be highly valued overall, although no statistically significant differences have appeared in the other dimensions.

In relation to the modality of the education, a clear trend is observed: the face-to-face learning and blended learning modalities are more likely to be highly rated by the professionals, in comparison with the e-learning modality, in all dimensions, except in the dimension of Organization and resources. In fact, e-learning activities show almost 4 times fewer probabilities than personal-attendance ones for being highly valued overall, and almost 3 times less than semi-personal-attendance ones. Semi-personal attendance activities are especially well valued with respect to those of the e-learning type in relation to Utility, Methodology used and Teaching capacity, whereas personal-attendance activities are better rated overall.

The duration, measurement via the numbers of hours entailed, and the time that the activity takes, measured in days, do not appear to have a significant lineal effect. Nevertheless, it is observed that those activities that are performed between 2 days and 2 weeks are 2.4 times less likely to be highly valued overall than those that only last for 1 or 2 days, irrespective of the number of hours that this may entail, or of their typology or modality.

Finally, when observing the effect of accreditation, the activities have almost 33% more probabilities to be highly valued in relation to their Utility, and 50% more in relation to their Methodology and to their Teaching capacity.

## Discussion

The sample used in this study consisted of 1,228 training activities, of which almost half were clinical conferences, just over a third were specialized courses, and the rest were learning workshops. The vast majority were face-to-face activities while one third were e-learning or blended learning. The Satisfaction with the Methodology dimension presented the highest correlations with the rest of the dimensions, placing it as a key dimension. All dimensions showed very high levels of satisfaction in general, with scores above 7.8 out of 10.

The results have demonstrated that the e-learning modality, which is only supported by new information and communication technologies (ICTs), systematically shows lower satisfaction levels compared to the two other modalities (face-to-face learning and blended learning) in the majority of the dimensions studied: Utility, Methodology and Teaching capacity.

The results presented herein are consistent with those found in previous studies that showed lower professionals’ levels of satisfaction with e-learning activities with regard to the methodology used^(^ 17 ^,^ 30 ^)^, the capacity of response by the teacher, and the suitability of the education for the educational needs of the professionals^(^ 5 ^,^ 30 ^)^. Nonetheless, those are advantages that were traditionally attributed both to online education and to semi-personal attendance education, or blended learning, such as flexibility^(^ 31 ^)^, anonymity in the platforms that facilitate participation, or the greater availability to be able to combine this with personal life and work^(^ 32 ^)^.

Recent studies found either low or very low evidence that e-learning education may have any effect on behavioural changes in health professionals or on patients’ outcomes^(^ 32 ^-^ 33 ^)^. Nevertheless, due to the continuous development of software tools that allow for online education to be carried out in a more personalized way, such as adaptive e-learning^(^ 34 ^)^, and the advantages that these courses allow for in support of educational self-management^(^ 17 ^)^, it may not be ruled out that further investigation in the subject area and further innovation in the training programs alignment with the real needs of professionals may improve the results obtained to date^(^ 6 ^)^.

Another of the most important findings is related to the differences found depending on the type of education. Previous works have shown the utility and acceptability of interdisciplinary clinical sessions or conferences in the health area^(^ 35 ^)^. Their organization, which is often of the personal-attendance form in the same health centres in which the professionals perform their work, as well as a methodology focused on very specific objectives, tend to be highly-valued aspects^(^ 36 ^-^ 37 ^)^. However, in spite of the importance of clinical conferences in the improvement of interdisciplinary communication^(^ 38 ^)^, there is a need to improve the pedagogical skills of teaching professionals^(^ 39 ^)^. The results obtained have reconfirmed both points since clinical conferences are more likely to be highly valued as regards their methodology and form of organization, but less in relation to the capacities of the teaching professionals.

In spite of the fact that, in her study, Hall found no solid evidence on the impact of the duration of the education - whether this may be measured in hours or spread over days - in relation to the satisfaction level perceived or to the acquisition of knowledge^(^ 40 ^)^, in the current study a slight trend has indeed been observed in the sense that the lower the number of hours, the greater the general satisfaction level, with such education being concentrated in a shorter period of time. The evidence has shown that the acquisition of knowledge by professionals in continuing education decreased slightly as the number of attendants at the course increased^(^ 41 ^)^. The results presented here sustain this hypothesis because, as the number of professionals in educational activity increases, the overall mean satisfaction levels with the utility, the methodology, and the teaching capacity, decrease.

Finally, the possible future effect of accreditation on the satisfaction of professionals was also considered. Accreditation of continuing education has become a basic tool to guarantee the quality of contents and methodology of continuing health education in the National Health System^(^ 2 ^)^. Despite its voluntary character, accreditation of continuing health education in the world has remained constant, after a slight decrease (7%) following the financial crisis^(^ 42 ^)^. No studies analysing the impact of accreditation of continuing health education on the satisfaction level of professionals have been found. However, different studies in other countries have demonstrated the satisfaction level of health professionals with health accreditation processes^(^ 43 ^)^. This study has shown a slight trend towards greater satisfaction perceived with accredited activities as compared with non-accredited ones, in particular with respect to utility, to the methodology used, and to the teaching capacity. Nevertheless, further investigation is required in relation to the effect of the accreditation of continuing health education on clinical results, the effectiveness of learning, and the satisfaction of professionals.

Some limitations of the study are worth mentioning: firstly, the data are based on mean scores of each one of the dimensions for each one of the educational activities that have participated in the survey, instead of using data from individual replies by professionals. Some eventual limitations in relation to the scope of the results that this may entail should be considered. However, those limitations have been sufficiently compensated by another two strong points; on one hand, the size of the sample and, on the other, the fact that not only one dimension of the satisfaction level has been evaluated but rather five.

Secondly, it may be asked why a logistic regression has been used instead of an ordinary least-squares regression (OLS) for continuous dependent variables such as those of satisfaction, on a scale of 0-10. The dependent variables did not fulfil even half of the most important suppositions that they must meet for OLS regression, that is, homoscedasticity, residual normality, autocorrelation, and non-collinearity. In order not to modify the data, they were transformed in binomial distribution.

## Conclusion

It can be concluded that the organizational, methodological, and resource-related characteristics that better predict the health professionals’ satisfaction with continuing education are the following, in this order: activities with a face-to-face learning modality, clinical conferences with trained teaching professionals, low number of hours, education programmes that are concentrated in short period of time, reduced number of professionals, and activities’ design based on quality criteria of accreditation models.

Lessons for the practice

- The e-learning modality must be designed with learning objectives more adapted to the expectations of learners and of limiting the scope of the learning to knowledge rather than to the acquisition of skills.
- While educational supports have progressed considerably in recent years, the methodological designs and the pedagogical capacities of teachers and professionals who present clinical conferences have not followed the same pace.
- A reduced teacher-student ratio is recommended in order to facilitate learning and its applicability to employment positions.

It is recommended to base the design of the education on quality criteria of accreditation models.

## References

[1] Accreditation Council for Continuing Medical Education CME Content: Definition and Examples.

[2] Muñoz-Castro FJ, Valverde-Gambero E, Villanueva-Guerrero L, Mudarra-Aceituno MJ, Vázquez-Vázquez M, Almuedo-Paz A (2012). Evolución de la formación continuada acreditada tras la puesta en
marcha de la Estrategia para la Seguridad del Paciente. Rev Calid Asist.

[3] Alford DP, Zisblatt L, Ng P, Hayes SM, Peloquin S, Hardesty I (2016). SCOPE of Pain : An Evaluation of an Opioid Risk Evaluation and
Mitigation Strategy Continuing Education Program. Pain Med.

[4] Aunión CD, Ruiz-Matas JH, Márquez AÁ, José Sánchez-Trincado Pavón M, Gil MA, Castro de la Nuez P (2019). Continuing Training Accreditation in the Organ Donation Process
in Andalusia: Results From the Education and Training Unit of the Regional
Transplant Organization of Andalusia. Transplant Proc.

[5] Allen LM, Palermo C, Armstrong E, Hay M (2019). Categorising the broad impacts of continuing professional
development: a scoping review. Med Educ.

[6] Rouleau G, Gagnon M-P, Côté J, Payne-Gagnon J, Hudson E, Dubois C-A (2019). Effects of E-Learning in a Continuing Education Context on
Nursing Care: Systematic Review of Systematic Qualitative, Quantitative, and
Mixed-Studies Reviews. J Med Internet Res.

[7] Liu Q, Peng W, Zhang F, Hu R, Li Y, Yan W (2016). The Effectiveness of Blended Learning in Health Professions:
Systematic Review and Meta-Analysis. J Med Internet Res.

[8] Mundet-Tuduri X, Crespo R, Fernandez-Coll ML, Saumell M, Millan-Mata F, Cardona A (2017). Expectations and perceptions of primary healthcare professionals
regarding their own continuous education in Catalonia (Spain): a qualitative
study. BMC Med Educ.

[9] American Dental Association (2017). American Dental Association's Continuing Education Recognition Program
(ADA CERP) recognition standards and procedures.

[10] American Medical Association (2017). The AMA Physician's Recognition Award and credit system.

[11] European Accreditation Council for Continuing Medical
Education (2016). EACCME Criteria For The Accreditation Of Live Educational Events
(LEE).

[12] Varetto T, Costa DC (2013). Continuing Medical Education Committee and
UEMS-EACCME. Eur J Nucl Med Mol Imaging.

[13] Varetto T, Costa DC (2014). The new UEMS-EACCME criteria for accreditation of live
educational events (LEEs): another step forward to improve the quality of
continuing medical education (CME) in Europe. Eur J Nucl Med Mol Imaging.

[14] Agencia de Calidad Sanitaria de Andalucía (2016). Manual de estándares de actividades de formación continuada (ME 3
3_03).

[15] Consejería de Salud, Junta de Andalucía (201). Estrategia de las Políticas de Formación del Sistema Sanitario Público
de Andalucía.

[16] Comisión de Formación Continuada de las Profesiones
Sanitarias (2013). Manual de procedimiento.

[17] Lawn S, Zhi X, Morello A (2017). An integrative review of e-learning in the delivery of
self-management support training for health professionals. BMC Med Educ.

[18] Baptista RCN, Martins JCA, Pereira MFCR, Mazzo A (2014). Students' satisfaction with simulated clinical experiences:
validation of an assessment scale. Rev. Latino-Am. Enfermagem.

[19] Hu A, Shewokis PA, Ting K, Fung K (2016). Motivation in computer-assisted instruction: Motivation in
CAI. Laryngoscope.

[20] Paliadelis PS, Stupans L, Parker V, Piper D, Gillan P, Lea J (2015). The development and evaluation of online stories to enhance
clinical learning experiences across health professions in rural
Australia. Collegian.

[21] European Accreditation Council for Continuing Medical
Education (2016). EACCME Criteria For The Accreditation Of E-Learning Materials
(ELM).

[22] United States Office of Personnel Management (2011). Training Evaluation Field Guide. Demonstrating the Value of Training at
Every Level.

[23] Kirkpatrick DL, Kirkpatrick JD (2007). Evaluación de acciones formativas: los cuatro niveles.

[24] Bijani M, Rostami K, Momennasab M, Yektatalab S (2018). Evaluating the Effectiveness of a Continuing Education Program
for Prevention of Occupational Exposure to Needle Stick Injuries in Nursing
Staff Based on Kirkpatrick's Model. J Natl Med Assoc.

[25] Rojo E, Maestre JM, Díaz-Mendi AR, Ansorena L, Del Moral I (2016). Innovation in healthcare processes and patient safety using
clinical simulation. Rev Calid Asist.

[26] Agencia de Calidad Sanitaria de Andalucía (2014). eValúa. Cuestionarios de evaluación de la formación.

[27] Esposito T, Muñoz-Castro FJ, Herrera-Usagre M, Periáñez-Vega M (2015). Fiabilidad y validez para un cuestionario de satisfacción con la
formación continuada en salud: el cuestionario de satisfacción del
discente. FEM.

[28] Agencia de Calidad Sanitaria de Andalucía Programa Integral para la acreditación de la Formación Continuada de las
profesiones sanitarias.

[29] Forsetlund L, Bjørndal A, Rashidian A, Jamtvedt G, O'Brien MA, Wolf F (2009). Continuing education meetings and workshops: effects on
professional practice and health care outcomes. Cochrane Database Syst Rev.

[30] McCutcheon K, O'Halloran P, Lohan M (2018). Online learning versus blended learning of clinical supervisee
skills with pre-registration nursing students: A randomised controlled
trial. Int J Nurs Stud.

[31] Vaona A, Banzi R, Kwag KH, Rigon G, Cereda D, Pecoraro V, Cochrane Effective Practice and Organisation of Care Group (2018). E-learning for health professionals. Cochrane Database Syst Rev.

[32] Sinclair PM, Kable A, Levett-Jones T, Booth D (2016). The effectiveness of Internet-based e-learning on clinician
behaviour and patient outcomes: A systematic review. Int J Nurs Stud.

[33] Voutilainen A, Saaranen T, Sormunen M (2017). Conventional vs. e-learning in nursing education: A systematic
review and meta-analysis. Nurse Educ Today.

[34] Fontaine G, Cossette S, Maheu-Cadotte M-A, Mailhot T, Deschênes M-F, Mathieu-Dupuis G (2019). Efficacy of adaptive e-learning for health professionals and
students: a systematic review and meta-analysis. BMJ Open.

[35] Maeda H, Tsujimura T, Yoshida K (2019). Questionnaire study on the utility of autopsy case conferences
related to emergency medicine practices. Medicine (Baltimore).

[36] Waldron MK, Washington SL, Montague GP (2016). Cooperative Clinical Conferences: Nursing Student Pediatric
Clinical Innovation. J Nurs Educ.

[37] Harrison Kelly S, Henry R, Williams S (2019). Using Debriefing Methods in the Postclinical
Conference. Am J Nurs.

[38] Prades J, Remue E, van Hoof E, Borras JM (2015). Is it worth reorganising cancer services on the basis of
multidisciplinary teams (MDTs)? A systematic review of the objectives and
organisation of MDTs and their impact on patient outcomes. Health Policy.

[39] Serçekus P, Baskale H (2016). Nursing students' perceptions about clinical learning environment
in Turkey. Nurse Educ Pract.

[40] Hall BM (2011). How Cognitive Requirement of Prompt and Time in Course Are Correlated
with Intersubjectivity within Threaded Discussions.

[41] Slater PJ, Herbert AR, Baggio S, Donovan LA, McLarty A, Duffield J (2018). Evaluating the impact of national education in pediatric
palliative care: the Quality of Care Collaborative Australia. Adv Med Educ Pract.

[42] ACCME (2017). ACCME Data Report Growth and Evolution in Continuing Medical Education -
2016.

[43] Brinza EK, Zhu LJ, Lilly M, Manning WJ, Needleman L, Gornik HL (2016). Accreditation is Perceived to Improve the Quality of Vascular
Testing Facilities. JVU.

